# Deep learning driven diagnosis of malignant soft tissue tumors based on dual-modal ultrasound images and clinical indexes

**DOI:** 10.3389/fonc.2024.1361694

**Published:** 2024-05-23

**Authors:** Haiqin Xie, Yudi Zhang, Licong Dong, Heng Lv, Xuechen Li, Chenyang Zhao, Yun Tian, Lu Xie, Wangjie Wu, Qi Yang, Li Liu, Desheng Sun, Li Qiu, Linlin Shen, Yusen Zhang

**Affiliations:** ^1^ Shenzhen Hospital, Peking University, Shenzhen, China; ^2^ College of Computer Science and Software Engineering, Shenzhen University, Shenzhen, Guangdong, China; ^3^ National Engineering Laboratory for Big Data System Computing Technology, Shenzhen University, Shenzhen, China; ^4^ West China Hospital, Sichuan University, Chengdu, Sichuan, China

**Keywords:** deep learning, artificial intelligence, ultrasound, soft tissue tumor, malignant tumor

## Abstract

**Background:**

Soft tissue tumors (STTs) are benign or malignant superficial neoplasms arising from soft tissues throughout the body with versatile pathological types. Although Ultrasonography (US) is one of the most common imaging tools to diagnose malignant STTs, it still has several drawbacks in STT diagnosis that need improving.

**Objectives:**

The study aims to establish this deep learning (DL) driven Artificial intelligence (AI) system for predicting malignant STTs based on US images and clinical indexes of the patients.

**Methods:**

We retrospectively enrolled 271 malignant and 462 benign masses to build the AI system using 5-fold validation. A prospective dataset of 44 malignant masses and 101 benign masses was used to validate the accuracy of system. A multi-data fusion convolutional neural network, named ultrasound clinical soft tissue tumor net (UC-STTNet), was developed to combine gray scale and color Doppler US images and clinic features for malignant STTs diagnosis. Six radiologists (R1-R6) with three experience levels were invited for reader study.

**Results:**

The AI system achieved an area under receiver operating curve (AUC) value of 0.89 in the retrospective dataset. The diagnostic performance of the AI system was higher than that of one of the senior radiologists (AUC of AI vs R2: 0.89 vs. 0.84, *p*=0.022) and all of the intermediate and junior radiologists (AUC of AI vs R3, R4, R5, R6: 0.89 vs 0.75, 0.81, 0.80, 0.63; *p <*0.01). The AI system also achieved an AUC of 0.85 in the prospective dataset. With the assistance of the system, the diagnostic performances and inter-observer agreement of the radiologists was improved (AUC of R3, R5, R6: 0.75 to 0.83, 0.80 to 0.85, 0.63 to 0.69; *p*<0.01).

**Conclusion:**

The AI system could be a useful tool in diagnosing malignant STTs, and could also help radiologists improve diagnostic performance.

## Highlights

- The deep-learning driven system has a high accuracy in diagnosing malignant soft tissue tumors.
- The deep-learning system showed superior performance than junior radiologists.
- The system is a useful tool for radiologists in discerning malignant soft tissue tumors.

## Introduction

Soft tissue tumors (STTs) are a group of superficial neoplasms with heterogeneous clinical manifestations and diverse pathological types. The ratio of benign to malignant is close to 100:1 (1). Soft tissue sarcomas are the most common malignant STTs, accounting for only 1% of all adult cancers (2). Despite the rarity of malignant STTs compared with other malignant entities, their hazards cannot be ignored due to the substantial mortality and morbidity (3, 4). The overall five-year survival rate of malignant STTs is about 50% (1). They also present high metastasis and postoperative recurrence rates, up to 39% for soft tissue sarcomas (5). Soft tissue sarcoma is one of the leading causes of death for young adults, particularly for certain subtypes (6). As a result, accurate diagnosis and timely treatment for malignant STTs is crucial to improve the prognosis of the patients. However, as STTs substantially vary in clinical manifestations, morphological changes, and biological behaviors, it is difficult to make precise classification of malignant STTs, which might lead to delayed diagnosis. It is reported that the diagnosis of soft tissue sarcoma was usually delayed for up to 94.6 weeks (7), which might cause disastrous consequences on patients’ outcome, such as a shorter survival time (8, 9).

Ultrasonography (US) is considered to be the first-line imaging method for STTs, due to its fast speed, high resolution, lower cost, availability, dynamic observation, and no contraindications (2). Gray-scale US can map the locations and morphological changes of STTs, including size, margin, shape, and internal components. The mobility, compressibility, and its anatomical associations with adjacent structures can also be confirmed by dynamic US. Color Doppler US can further display the distribution of intra-tumoral and peri-tumoral blood vessels (10–14). However, US presented an unstable and relatively low diagnostic performance for classifying benign and malignant STTs. The reported accuracy rate of US varied among studies, ranging from 69% to 93% (10–12, 15, 16). And most of the previous studies only involved a small number of cases for evaluation, compromising their reliability. Meanwhile, conventional US is characterized by high operator-dependence and relatively low inter-observer agreement, which also degrade its performance in classifying malignant STTs. How to improve the diagnosis accuracy of US for malignant STTs, at the same time decrease operator dependence, is a very important research topic.

Advanced techniques like Artificial intelligence (AI), especially deep learning (DL) algorithms, possess an excellent ability in image recognition tasks. DL is emerging as a promising tool to resolve various radiology tasks using US images, including screening breast cancer (17, 18), classifying thyroid nodules (19–21), diagnosing liver diseases (22–24), and assessing musculoskeletal abnormality (25). Apart from showing good diagnostic performances in some diseases, DL can also assist radiologists in enhancing their accuracy and reliability in reading US images (26). Currently, AI-based malignant STTs diagnosis based on US images is still in the initial stage (27–29). These studies applied non-DL methods to develop assistant tools for malignant STTs diagnosis, with limited enrolled STTs cases. And clinical indexes were not fully utilized for the model construction in the previous studies.

To overcome the barrier of US diagnosis of malignant STTs, we established this DL-driven AI system, named ultrasound clinical soft tissue tumor net (UC-STTNet), for predicting STTs based on US images and clinical indexes of the patients. First, one of the highlights of the study is the application of two modalities of US imaging, gray-scale US and color-Doppler US, in model construction, which could provide more morphological information of STTs masses. And basic clinical indexes were also incorporated in the system for a more comprehensive diagnosis of the tumors. Also, we used 5-fold cross validation method in the model building based on a large database. The AI system could also provide heatmaps of US images illustrating the features relevant to model predictions for radiologists to make diagnosis. The AI system could successfully improve the performances and stability of the radiologists in classifying malignant STTs. To the best of our knowledge, our work is the first one applying DL technology for US diagnosis of malignant STTs.

## Materials and methods

### Ethical approval

The study was designed as a retrospective study and approved by the ethics committee of Peking University Shenzhen Hospital (Approval number: 202200901). The informed content was not waived since the retrospective study was observational and did not involve any interventional procedures. And all the information of the patients is anonymized throughout the study. The ethics committee approved the omission of informed content.

### Study participants enrollment

In this work we employed five-fold cross validation for network evaluation. Among 5 folds, 4 and 1 folds were employed for training and testing, respectively. To build the training and testing dataset, we retrospectively reviewed the clinical and imaging data of the patients with STTs from July 2013 to December 2021. The patients with dual-modal US images and pathological results from surgical resections or biopsies were enrolled. To further evaluate the performance of the AI system, we collected a prospective testing dataset from April 2022 to September 2022 in our hospital. Tumors that occurred in superficial organs, including thyroid gland, breast, salivary gland, and lymph nodes, were excluded in both of the retrospective and prospective workflows.

### US imaging and clinical data collection

All US images were derived from US imaging database at Peking University Shenzhen Hospital. The US examinations were performed by radiologists with over five-year experiences of US using commercial US equipment with 5-15MHz probes. Two representative pictures of each patient, one gray scale image showing the largest section of tumor and one color Doppler flow image with the most abundant blood vessels, were selected for model building. The US images of STTs were reviewed and selected from the patients by two radiologists with five-year experiences in US together for image quality control. When disagreement occurred between the two radiologists, they would refer to a third radiologist with over 10-year experiences for the final decision. Two clinicians collected the clinical data for the enrolled patients, including sex, age, duration, locations, layer, the maximum and minimum diameter of lesions, depth from skin, history of malignancy, and surgical history.

### DL architecture development

We designed a multi-data fusion convolutional neural network, named as UC-STTNet, to combine gray scale and color Doppler US images and clinic features for malignant STTs diagnosis. Detailed descriptions about UC-STTNet are shown in Supplementary materials (**Supplementary 1**; **Supplementary Figure 1**; **Supplementary Table 1**).

The image feature extraction consisted of a tumor area enhancement block and a tumor feature extraction block. The tumor area enhancement block was an encoder-decoder network, which employed ResNet18 as backbone and with five down- and up-sample layers. The encoder was employed to extract the region of interest (ROI) feature of STTs, and the decoder was used to generate a ROI feature map which represented the possibility of tumor area (abbr. ROI-map).

The clinical data was directly digitized as a feature vector, which was then processed by a multi-layer perceptron and directly input into the multi-data fusion block. The multi-data fusion block consisted of feature concatenation and attention mechanism. The segmentation and tumor area features were concatenated together, and then the combined features were input into an attention block.

Global average pooling was used to align the image features to linear space and then concatenated with the features of clinic data to generate a multi-data fusion feature for the final STTs classification. Gradient-weighted Class Activation Mapping (Grad-CAM) was adopted in the classification tasks on deep learning to explain the performance of the proposed UC-STTNet. And we used a weighted combination for the forward activation map and activated the result by Rectified Linear Activation function (ReLU) to get the visualization heatmap.

### Reader study and AI-assisted reader study

Six radiologists with three experience levels were invited to review the dual-modal US images and clinical manifestations independently and make diagnosis. The six radiologists participated the reader study included two senior radiologists with 21 and 24 years of experience (R1 and R2), two intermediate radiologists with 10 and 12 years of experience (R3 and R4), and two junior radiologists with 4 and 7 years of experience (R5 and R6). The radiologists were blind to the pathologic results of the tumors. One month after the original reader study, the same STTs cases were re-presented to the six radiologists for a second diagnosis, along with the AI-predicted results and heatmaps as reference. The radiologists were blind to their first-time results and pathological results of the tumors.

### Statistical analysis

The 5-fold cross validation was used for model training and testing. The split was randomly repeated for five times and the average performances were recorded. The receiver operating curve (ROC), area under ROC curve (AUC), accuracy, sensitivity, specificity, positive predictive value (PPV) and negative predictive value (NPV) with 95% confidence interval (CI) were used to evaluate the diagnostic performance of the model, the radiologists, and the radiologists with AI assistance. AUC values of the same dataset and different datasets were compared to use the methods reported by DeLong et al (30) and Hanley and McNeil (31), respectively. We further calculated the intra-class correlation (ICC) with 95% CI to evaluate the inter-observer variability of the six radiologists before and after the assistance of AI results. *P* < 0.05 was considered as statistically significant. The statistical analyses were performed by using Medcalc (Version 20.0, MedCalc Software Ltd, Belgium).

## Results

**Figure 1:**
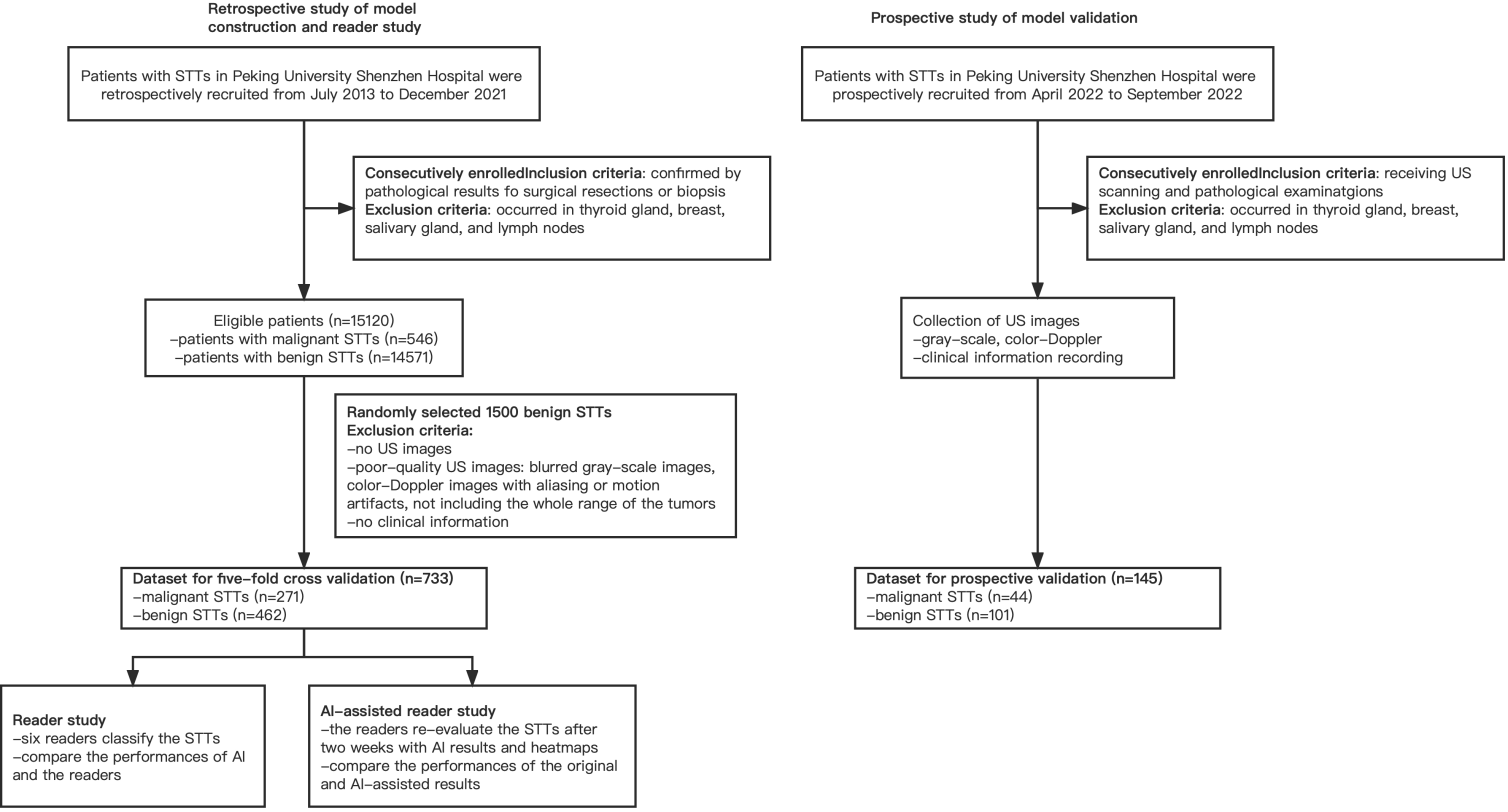
Overall study flow of UC-STTNet, the AI system for STTs diagnosis. The AI system was developed on a deep learning frame work using the tumor information from both dual-modal US images, including gray-scale US and color-Doppler US, and clinical features. The AI system could help radiologists in clinical decision-making by providing prediction results of STTs and heatmaps of US images as reference.

In this study, we developed and presented UC-STTNet, an AI system based on a deep-learning architecture for malignant STTs diagnosis. The study flow of the construction and validation of UC-STTNet is shown in **Figure 1**.

**Figure 2:**
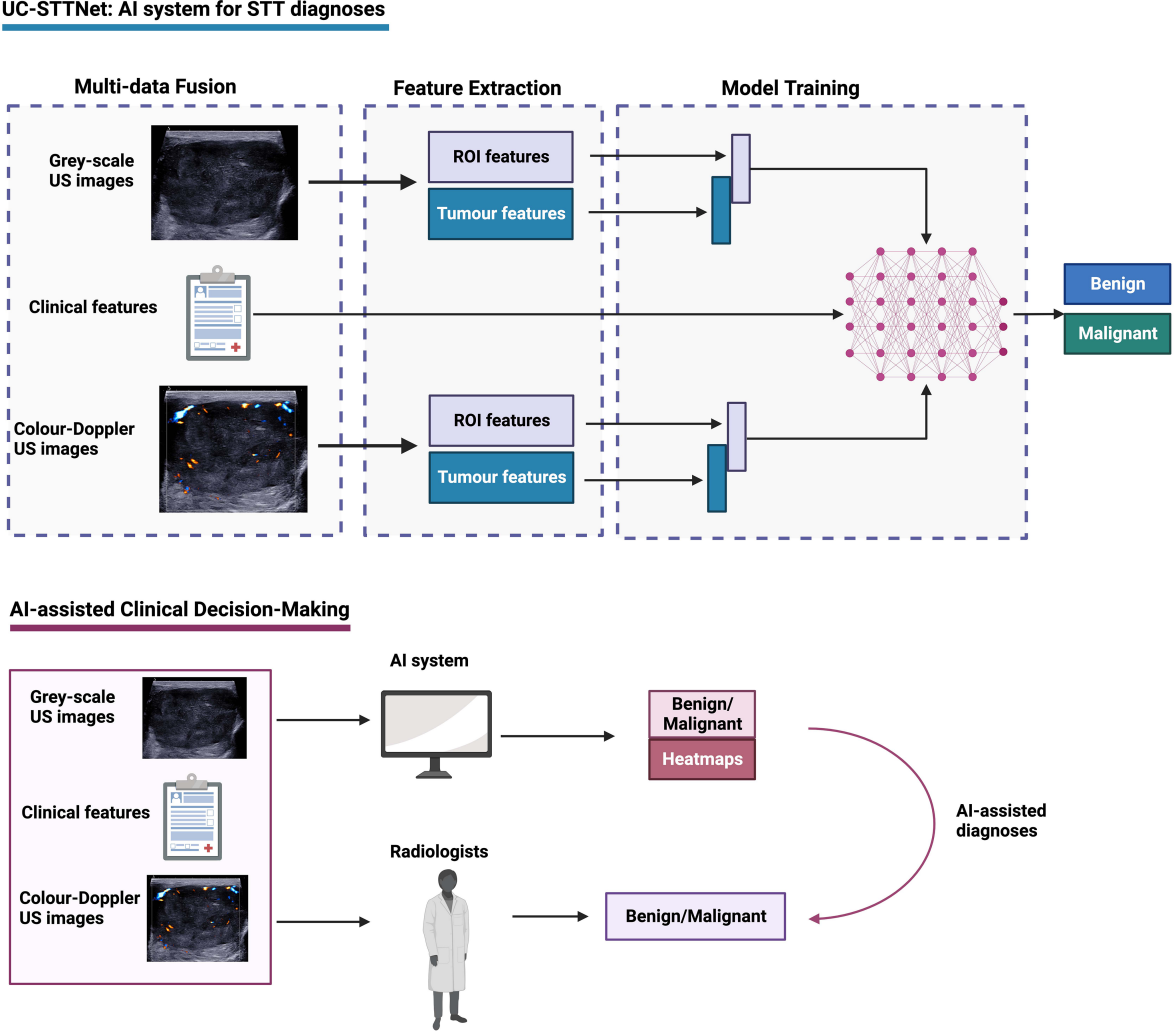
Flow chart of the retrospective and prospective patients’ recruitment.

From July 2013 to December 2021, a total of 15120 adult patients with STTs were received from pathology data, which came from core biopsy or surgery. There were 546 patients with malignancy and 14574 patients with benign. Finally, 733 masses of 693 patients, including 271 malignant masses of 231 patients and 462 benign masses of 462 patients, were enrolled in this study to build the model. For the prospective validation of the model, we recruited 145 masses of 145 patients, including 44 malignant masses of 44 patients and 101 benign masses of 101 patients from April 2022 to September 2022. The workflow of the retrospective and prospective patient recruitment is shown in **Figure 2**.

### Clinical data and pathological results

The clinical features and pathological results of the retrospectively enrolled and prospectively enrolled STTs were listed in **Table 1**. Except sex, the other clinical characteristics between benign and malignant masses, were significantly different.

**Table 1:** Clinical characteristics of 733 retrospective STTs masses and 145 prospective STTs masses.

	AUC	Accuracy	Sensitivity (%)	Specificity (%)	PPV	NPV
AI system	0.89 (0.87-0.92)	0.84 (0.82-0.87)	0.76 (0.71-0.81)	0.90 (0.87-0.92)	0.81 (0.76-0.86)	0.86 (0.83-0.89)
R1	0.87 (0.85-0.90)	0.88 (0.86-0.91)	83.4 (78.4-87.6)	91.34 (88.4 - 93.7)	0.85 (0.81 ~ 0.89)	0.90 (0.88 ~ 0.93)
R1+AI	0.88 (0.86-0.91)	0.89 (0.87-0.91)	86.4 (81.7-90.2)	90.26 (87.2 - 92.80)	0.84 (0.79 ~ 0.88)	0.92 (0.89 ~ 0.94)
R2	0.84 (0.81-0.87)	0.87 (0.85 -0.89)	72.7 (67.0-77.9)	95.24 (92.9 - 97.0)	0.90 (0.86 ~ 0.94)	0.86 (0.83 ~ 0.89)
R2+AI	0.82 (0.79-0.85)	0.86 (0.83-0.89)	68.3 (62.4-73.8)	96.32 (94.2 - 97.8)	0.92 (0.88 ~ 0.95)	0.84 (0.81 ~ 0.87)
R3	0.75 (0.72-0.78)	0.80 (0.78-0.83)	54.6 (48.5-60.6)	95.45 (93.1 - 97.2)	0.88 (0.83 ~ 0.93)	0.78 (0.75 ~ 0.82)
R3+AI	0.83 (0.80-0.86)	0.85 (0.82-0.88)	75.3 (69.7-80.3)	90.69 (87.7 - 93.2)	0.83 (0.78 ~ 0.87)	0.86 (0.83 ~ 0.89)
R4	0.81 (0.78-0.83)	0.82 (0.79-0.85)	76.1 (70.5-81.0)	85.06 (81.5 - 88.2)	0.75 (0.70 ~ 0.80)	0.86 (0.83 ~ 0.89)
R4+AI	0.81 (0.78-0.84)	0.83 (0.81-0.86)	71.6 (65.8-76.9)	90.04 (86.9 - 92.6)	0.81 (0.76 ~ 0.86)	0.84 (0.81 ~ 0.88)
R5	0.80 (0.77-0.83)	0.80 (0.77-0.83)	83.4 (78.4-87.6)	77.27 (73.2 - 81.0)	0.68 (0.63 ~ 0.73)	0.89 (0.86 ~ 0.92)
R5+AI	0.85 (0.82-0.88)	0.86 (0.84-0.89)	80.07 (74.8-84.7)	90.04 (86.9 - 92.6)	0.83 (0.78 ~ 0.87)	0.89 (0.86 ~ 0.91)
R6	0.63 (0.59-0.66)	0.71 (0.68-0.74)	31 (25.5-36.9)	94.16 (91.6 - 96.1)	0.76 (0.68 ~ 0.84)	0.70 (0.66 ~ 0.74)
R6+AI	0.69 (0.65-0.72)	0.76 (0.73-0.79)	40.22 (34.3-46.3)	96.97 (95.0 - 98.3)	0.89 (0.83 ~ 0.94)	0.73 (0.70 ~ 0.77)

STTs, soft tissue tumors.

### Performance of the AI system on the retrospective dataset for model building

The performance of the AI system was evaluated using 5-fold cross validation. Of the five validation sets, the highest AUC was 0.91 (95% CI: 0.84, 0.95), with accuracy of 0.89 (95% CI: 0.84, 0.94), sensitivity of 0.82 (95% CI: 0.72, 0.82), specificity of 0.93 (95% CI: 0.88, 0.98), PPV of 0.88 (95% CI: 0.79, 0.97), NPV of 0.90 (95% CI: 0.84, 0.96), respectively. The average AUC, accuracy, sensitivity, specificity, PPV and NPV of the model in the five validations were 0.89 (95% CI: 0.87, 0.92), 0.84 (95% CI: 0.82, 0.87), 0.76 (95% CI: 0.71, 0.81), 0.90 (95% CI: 0.87, 0.92), 0.81 (95% CI: 0. 76, 0.86) and 0.86 (95% CI: 0.83, 0.89), respectively. The AI system showed higher specificity than sensitivity, indicating that the majority of the benign cases (above 90%) were accurately recognized. While there were around 20% of the malignant cases mistakenly classified as benign. Similarly, the NPV value of the model was slightly higher than the PPV value, indicating that the AI system had more confidence (around 3%) in predicting benign cases.

**Figure 3:**
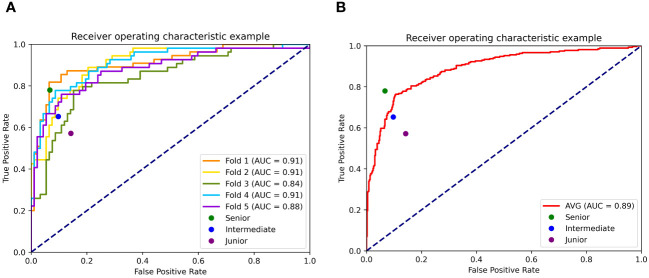
Receiver operating characteristic curves (ROC) of UC-STTNet assessed by 5-fold cross validations and comparing the different level radiologists. 3 **(A)**. ROC of each fold of the AI system and three different levels of radiologists; 3 **(B)**. the average performance of the AI system compared with three levels radiologists.

**Figure 3A** presents the ROCs of all five folds. According to the figure, UC-STTNet appears to be robust and stable when trained and tested with different folds of data. The AUC values of the AI system in the five validations ranged from 0.84 to 0.91, with the standard deviation 0.028. **Figure 3B** depicted the performances of radiologists with three different experience levels. The diagnostic performance of UC-STTNet was higher than that of one of the senior radiologists (AUC of UC-STTNet vs AUC of R2: 0.89 vs. 0.84, *p*=0.022) and all the intermediate and junior radiologists (AUC of UC-STTNet vs AUC of R3, R4, R5, and R6: 0.89 vs 0.75, 0.81, 0.80, 0.63; *p <*0.01), and was comparable to one of the high-level radiologists (AUC of UC-STTNet vs AUC of R1: 0.89 vs 0.87, *p*=0.30). And there were no significant differences in accuracy, specificity and PPV between the AI system and the intermediated-level radiologists (*p*=0.09, 0.96, and 0.72, respectively). And the AI system showed better sensitivity and NPV than the intermediated-level radiologists (*p*=0.01 and 0.04, respectively).

### Assistant role of the AI system for radiologists

**Figure 4:**
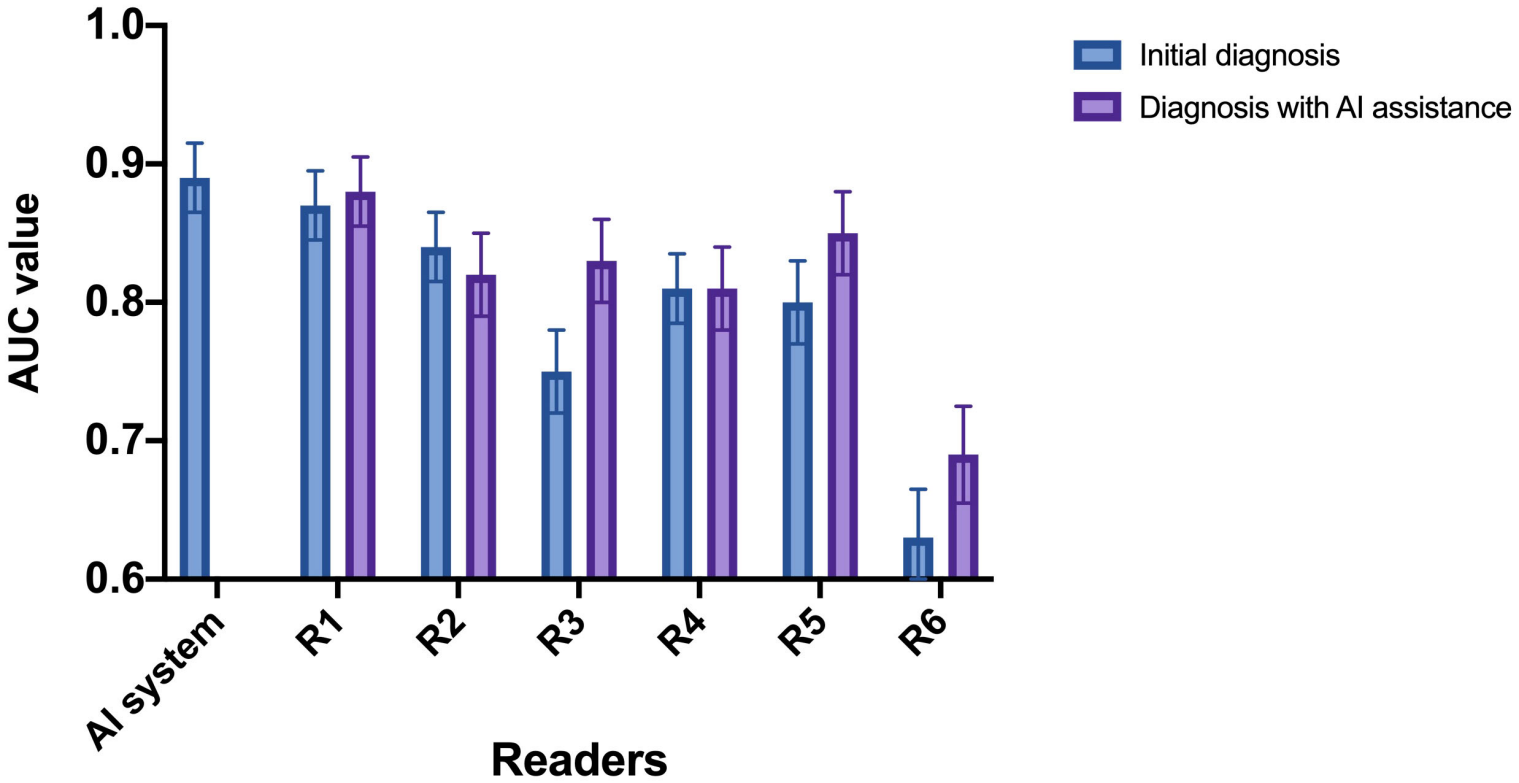
AUC of the radiologists with and without referring to the AI system. R1 and R2: senior radiologists; R3 and R4: intermediate radiologists; R5 and R6: junior radiologists. For junior radiologist (R5 and R6) and one of the intermediate radiologists (R3), the AUC after the AI assistance were significantly improved.

. The change in diagnostic performance of each radiologist after the assistance from the AI system was displayed in **Figure 4**l **Table 2**. For junior radiologists (R5 and R6) and one of the intermediate radiologists (R3), the AUC values after the AI assistance were significantly improved (R3: 0.75 to 0.83, *p*<0.01; R5: 0.80 to 0.85, *p*<0.01; R6: 0.63 to 0.69, *p*<0.01), indicating that the diagnostic performances of the radiologists could be enhanced via the aid of the AI system.

**Table 2:** Performance of the AI system, the radiologists with three experience levels, and AI-assisted radiologists.

Clinical Characteristics	Retrospective STTs masses				Prospective STTs masses			
	Overall	Benign masses	Malignant masses	P-Value	Overall	Benign masses	Malignant masses	P-Value
Sex				0.8825				0.100
Male	338	214	124		61	38	23	
Female	395	248	147		84	63	21	
Age	27.6±15.4	40.3±13.9	51.7±15.3	<0.001	41.96±15.5	38.8±13.4	49.2±17.6	0.057
Malignant History				<0.001				<0.001
Yes	164	17	147		13	0	13	
None	579	445	124		132	101	31	
Surgical History								
Yes	189	63	126	<0.001	12	101	32	<0.001
None	544	399	146	<0.001	133	0	12	
Tumor Duration (months)	27.6±47.8	33.9±52.1	15.5±42.2	<0.001	28.3±42.8	31.4±46.2	21.9±34.7	0.06
Tumor Position				<0.001				<0.001
Head or neck	118	91	27		39	30	9	
Truck	286	109	177		24	10	14	
Upper limb	193	172	21		48	43	5	
Lower limb	136	90	46		34	18	16	
Tumor Side				0.012				0.688
Left	303	186	117		67	49	18	
Right	342	208	134		58	39	19	
Mid	88	68	20		20	13	7	
Tumor Long Diameter (mm)	33.2±29.4	25.9±19.4	45.7±38.1	<0.001	34.7±38.5	22.8±15.6	62.3±57.3	0.026
Tumor Short Diameter(mm)	16.1±16.2	11.4±10.8	24.2±20.2	<0.001	16.3±20.7	9.5±8.0	32.0±30.4	<0.001
Tumor Depth(mm)	4.5±4.2	3.6±3.3	6.2±4.9	<0.001	4.3±5.0	2.8±2.1	7.8±7.5	0.005
Anatomical Level				<0.001				
Superficial fascia layer	613	412	201		113	95	18	<0.001
Deep fascia layer	120	50	70		32	6	26	
Pathological types	Retrospective STTs masses				Prospective STTs masses			
Malignant types	271				44			
Sarcoma	74				33			
Metastasis	137				0			
Lymphoma	13				3			
Squamous-cell carcinoma	13				5			
Melanoma	9				3			
Others	25				0			
Benign types	462				101			
lipoma	109				22			
hemangioma	57				20			
epidermoid cyst	51				10			
schwannoma	35				8			
giant cell tumor	25				0			
Others	185				41			

AI, Artificial intelligence; R1 and R2: senior radiologists R3 and R4: intermediate radiologists R5 and R6: junior radiologists.

Subsequently, we calculated the ICC value among the six radiologists in classifying the malignant STTs. The original ICC value of the radiologists before referring to the AI system was 0.87 (0.84-0.89), which increased to 0.92 (0.91-0.93) after AI assistance, indicating the diagnostic agreement of the radiologists could be improved via the aid of the AI system.

### Explainability of the AI system

**Figure 5:**
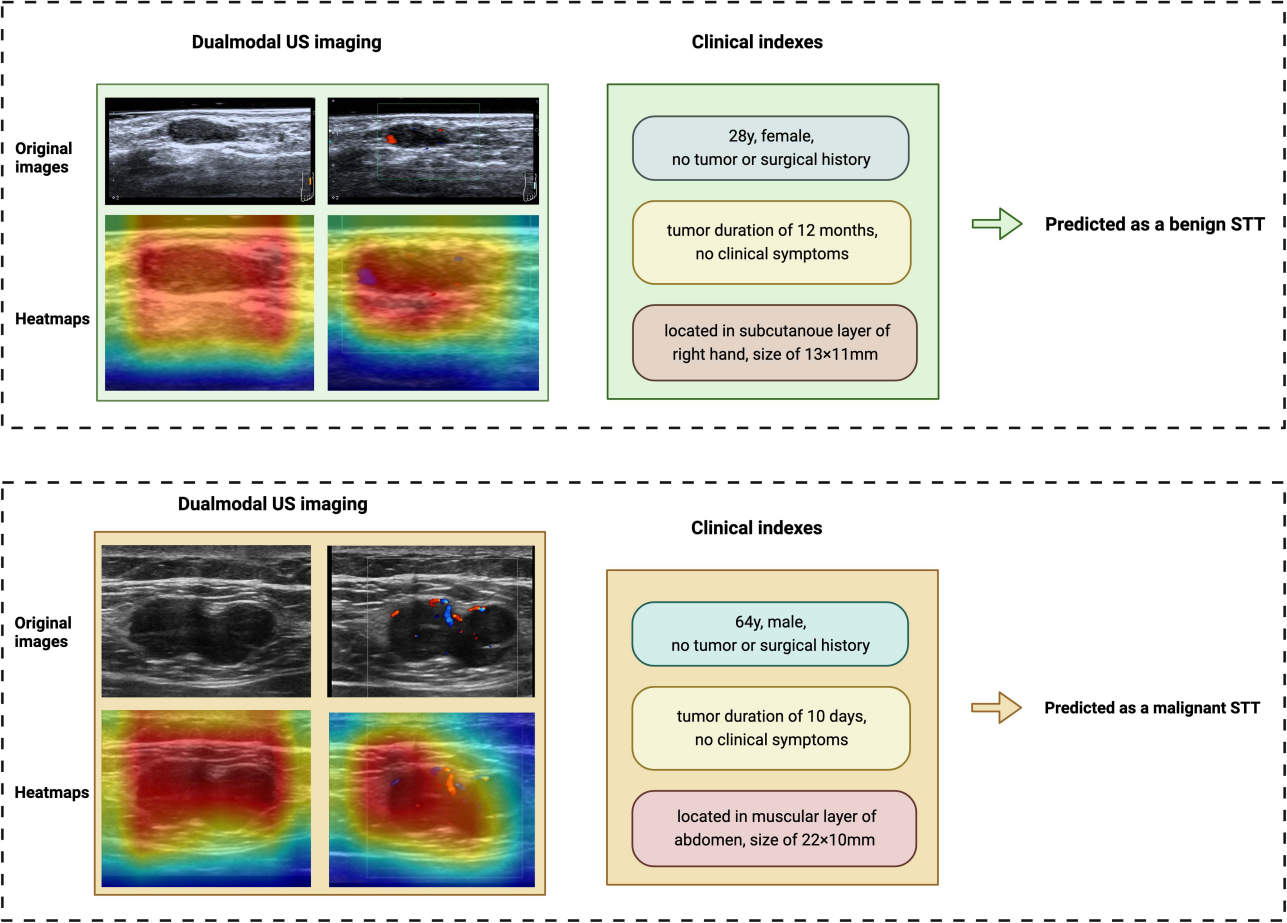
Examples of the AI system classifying benign and malignant STTs. The AI system diagnosed STTs based on dual-modal US images and clinical features. Heatmaps of the two modalities of US were also provided by the system. The above case is a 28-year-old female with a STTs mass on the subcutaneous layer of the right hand. She had no tumor or surgical history. The tumor was found 12 months ago and had a size of 13×11mm. The AI system diagnosed it as a benign tumor, which was identified as a benign schwannoma by pathology. The other case is a 64-year-old male with a STTs mass on the muscular layer of abdomen. The patient also reported no tumor or surgical history. The tumor was found 10 days ago and had a size of 22×11mm. The AI system diagnosed it as a malignant STTs tumor, which was identified as a metastatic malignant melanoma by pathology.

Explainability of UC-STTNet was demonstrated as heatmaps that highlights the significant areas attended by the model for malignant STTs diagnosis. The examples of the AI prediction of malignant STTs were illustrated in **Figure 5**. UC-STTNet gave the prediction result of a malignant STTs mass based on its dual modal US images and clinical indexes. The heatmap of the mass was generated by the AI system and used as reference for radiologists.

### Performance of the AI system on the prospective dataset

**Figure 6:**
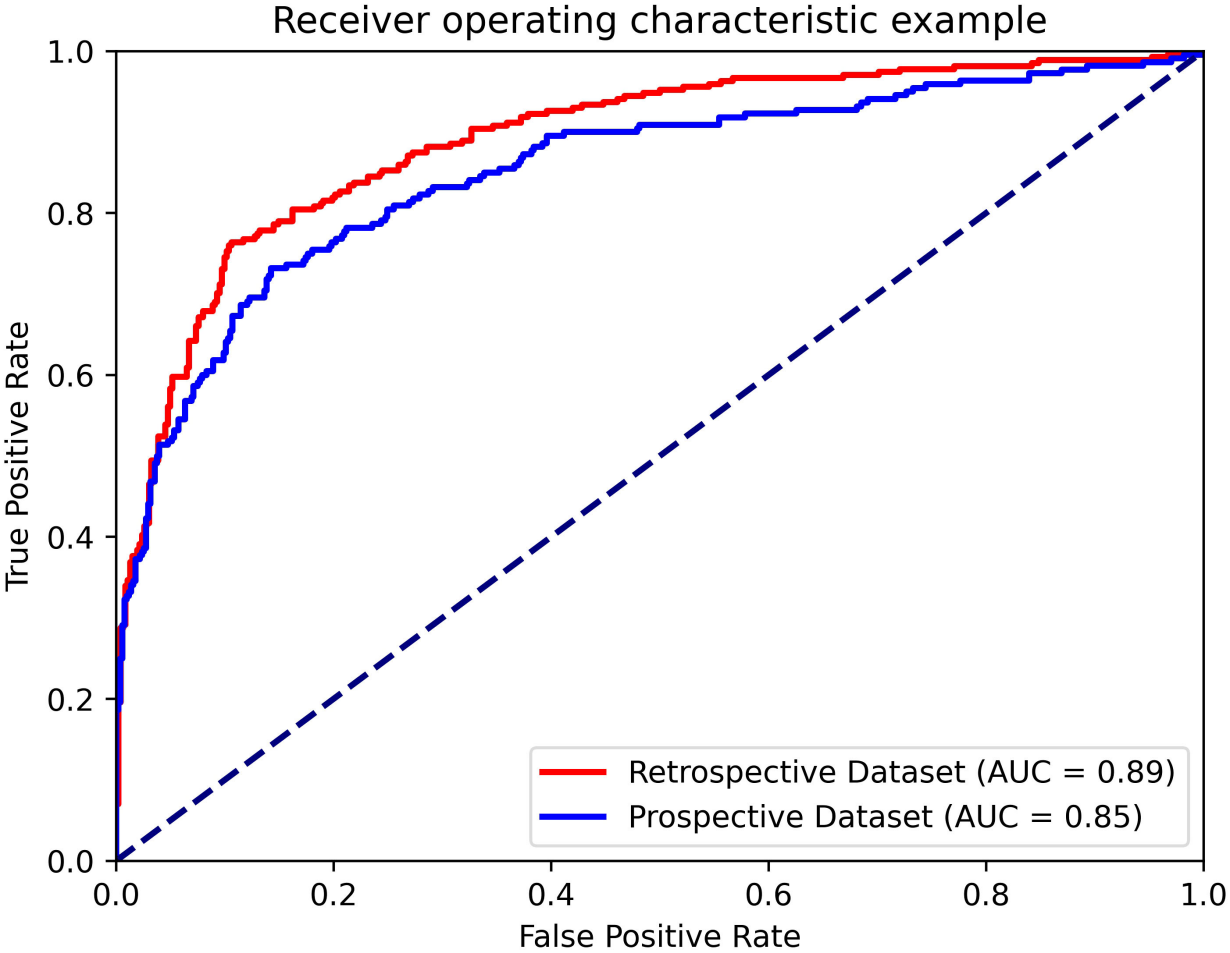
ROC curves of the AI system on the retrospective and prospective datasets.

The AUC, accuracy, sensitivity, specificity, PPV and NPV of the AI system on the prospective dataset were 0.85 (95% CI: 0.82, 0.89), 0.83 (95% CI: 0.77, 0.90), 0.63 (95% CI: 0.49, 0.78), 0.91 (95% CI: 0.86, 0.97), 0.75 (95% CI: 0.62, 0.90) and 0.85 (95% CI: 0.79, 0.92), respectively. The AUC value of the AI system on the prospective dataset had no statistical difference with the average AUC value on the model-building dataset (0.89 vs 0.85, *p*=0.282). The diagnostic performance of the AI system in the prospective dataset is shown in **Figure 6**.

## Discussion

In this study, a total of 733 and 145 masses were collected retrospectively and prospectively. We successfully built a DL-driven AI system, named UC-STTNet, for distinguishing the malignant STTs from benign ones based on dual modal US images and clinical manifestations. The AI system achieved the average AUC value of 0.89 in the retrospective dataset, showing a diagnostic performance comparable to high-level radiologists, superior to intermediate and junior radiologists. With the assistance of the system, the diagnostic performances and inter-observer agreement of the radiologists could be further enhanced. To note, the number of STTs patients and cases involved in our study is so far the largest, among all available literature works.

Diagnostic models for classifying malignant STTs based on US images have been developed by several studies. Despite of their high accuracy, the previous models have some disadvantages and are not suitable for clinical promotion. Chen et al. developed a computer-aid-diagnosis (CAD) system using US images to improve the accuracy of 89.5% for malignant STTs (27). However, manual identifications of lesions were required in their CAD system, which is time-consuming and not convenient for clinical application. Wu MJ et al. established a STTs diagnostic nomogram integrating ultrasound and clinical features via multivariable regression analysis, which achieved an AUC value of 0.896 (29). The sample size for model building was also relatively small. Compared with previous studies, our study has the following strengths. Firstly, we used deep learning algorithm to develop the AI system for diagnosis, which was more intelligent and robust than the hand-crafted systems, and could made automatic diagnosis of the masses. Secondly, the AI system utilized the imaging data of two US modalities, the gray scale and color Doppler US, as well as clinical information, to make a more comprehensive diagnosis of the tumors. Also, the AI system was built on a relatively large number of cases, and its accuracy and robustness were validated on a prospective dataset. The diagnostic performance of UC-STTNet was comparable with a meta-analysis of elastography in assessment of malignant STTs (16). The average AUC and accuracy of our system were 0.89 and 0.84, demonstrating a better performance than the contrast-enhanced ultrasound (CEUS) for predicting the malignancy of STTs, whose AUC and accuracy were 0.86 and 0.81, respectively (32).

We also verified the assistant role of the AI system for radiologists in making diagnosis of malignant STTs. While our results showed that UC-STTNet was superior to the performance of intermediate and junior radiologists, our AI system could help these less experience radiologists make more accurate diagnosis. Meanwhile, the inter-observer agreement of the radiologists was also improved when they referred to the diagnostic results of the AI system. UC-STTNet not only provided the final predictive results of the masses, but also generated heatmaps representing the active areas for diagnosis for the radiologists. Therefore, the AI system could be utilized as an assistant tool for the radiologists to enhance their diagnostic performance and stability in STTs, as well as to decrease operator dependence. To note, compared with other models for diagnosing STTs, including the model based on hand-crafted ultrasound features and the model based on radiomics, the process of using our AI system is more clinical applicable. The AI system can directly generate the result for prediction and does not need lesion delineation and feature extraction. For further clinical promotion of the AI system in the future, we will attempt to integrate the DL architecture into commercial US devices as an on-board software to help to improve the diagnosis performance and decrease workforce for radiologists.

The AI system tends to misdiagnose the benign masses with large size, usually more than 30mm in longitude. The benign STTs that possessing abundant blood vessels on color Doppler US imaging, such as glomangioma, could also be misdiagnosed by the system. On the other hand, the malignant tumors with small size and scarce vasculature might be classified as benign ones. In addition, a total of 6 cases of dermatofibrosarcoma protuberans (DFSP) were predicted as benign by the AI system. For DFSPs, skin changes should also be taken into account during diagnosis. Additionally, misdiagnosis often occurs in patients with a history of malignancy. To prevent the aforementioned misdiagnosis scenarios, more cases should be supplied for model development in the future study. **Supplementary Figure 2** demonstrated the examples of the misdiagnosed STTs cases of the AI system.

Our study has several limitations. First, it was a single center research. The AI system was not verified by external validation from multi-center datasets. The sensitivity of the AI system was relatively low in the prospective validation dataset. which should be improved by enrolling more malignant cases in the training dataset in further studies. Also, we only used two modalities of US images to build the model, and other available US modalities, including US elastography and CEUS, were not incorporated in our study. The two US modalities will be added to the system in our future study to improve its diagnostic accuracy. Moreover, we compared the performance of the DL model with the radiologists on the retrospective dataset due to its relatively large sample size. In the future study, the accuracy of the model will be further explored on a large prospective data.

## Conclusions

A DL-driven AI system based on dual-modal US images and clinical features for malignant STTs diagnosis was developed on a retrospective dataset of STTs. It achieved a high accuracy in predicting malignant STTs on both retrospective and prospective datasets. The performance of the AI system was comparable to senior radiologists, and better than junior and intermediate radiologists. The developed AI system could also assist radiologists in improving their diagnostic accuracy and stability in classifying malignant STTs.

## Data availability statement

The raw data supporting the conclusions of this article will be made available by the authors, without undue reservation.

## Ethics statement

The studies involving humans were approved by the ethics committee of Peking University Shenzhen Hospital. The studies were conducted in accordance with the local legislation and institutional requirements. Written informed consent for participation was not required from the participants or the participants’ legal guardians/next of kin in accordance with the national legislation and institutional requirements.

## Author contributions

HX: Writing – review & editing, Supervision, Project administration. YZ: Writing – original draft, Software, Formal analysis. LD: Writing – original draft, Software, Methodology, Data curation. HL: Writing – original draft, Software, Formal analysis, Data curation. XL: Writing – original draft, Software, Methodology, Formal analysis. CZ: Writing – original draft, Software, Formal analysis, Data curation. YT: Writing – original draft, Data curation. LX: Writing – original draft, Data curation. WW: Writing – original draft, Data curation. QY: Writing – original draft, Data curation. LL: Writing – review & editing, Supervision. DS: Writing – review & editing, Supervision. LQ: Writing – review & editing, Supervision. LS: Writing – review & editing, Supervision, Project administration. YZ: Writing – original draft, Data curation.
