# Supplementary material for: Deep learning driven diagnosis of malignant soft tissue tumors based on dual-modal ultrasound images and clinical indexes
Source: Front Oncol. 2024 May 23;14:1361694. doi: 10.3389/fonc.2024.1361694 (PMC11153704; doi:10.3389/fonc.2024.1361694)
Supplement: Supplementary file 1 [file DataSheet_1.docx]

**Supplementary 1. Detailed methods of deep-learning architecture development**

**Image data preprocessing**

All images were uniformly resized to 256×256 pixels for training and testing. Considering the influence of overfitting in our relatively small-scaled dataset, in training process we employed the data augmentation strategies, including random vertical and horizontal flip, rotation within ±15 degrees, and adding gaussian noise with $\mu=0$and $\sigma^{2}\in[0.1, 0.9]$.

**Network structure of UC-STTNet:** In the multi-data fusion convolutional neural network (UC-STTNet), both gray scale and color US images were input into a twin-branch image feature extraction network for ROI and tumor feature extraction, where both branches had the same structure. A multi-data fusion block was developed for combining both image and clinic features.

**Image feature extraction:** The image feature extraction consisted of a tumor area enhancement block and a tumor feature extraction block. The tumor area enhancement block was an encoder-decoder network, which employed ResNet18 as backbone and with five down- and up-sample layers. The encoder was employed to extract the ROI feature of STTs, and the decoder was used to generate a ROI feature map which represented the possibility of tumor area (abbr. ROI-map). To emphasize the foreground area and reduce the influence of the background, we re-ranged the distribution of the ROI-map from [0,1] to [0.5,1.5], and then employed a pixel-wised product operation between original image input and the generated ROI-map to filter out the irrelevant area, and emphasize the tumor area of the original US images simultaneously. After emphasizing the tumor area, we employed a tumor feature extraction block, with two inception layers and three convolution layers, to extract tumor features from the processed images.

**Clinic features extraction:** The clinical data was directly digitized as a feature vector, which was then processed by a multi-layer perceptron and directly input into the multi-data fusion block.

**Multi-data fusion:** The multi-data fusion block consisted of feature concatenation and attention mechanism. The segmentation and tumor area features were concatenated together, and then the combined features were input into an attention block (SE block was employed in this work), which evaluated the importance of each channel of concatenated feature and assigned a higher weight to the more significant feature and a lower weight to an opposite one. Finally, global average pooling was used to align the image features to linear space and then concatenated with the features of clinic data to generate a multi-data fusion feature for the final STT classification.

**Classification:** Here we employed clinical encoder and image feature compress to balance the influence of the clinical and image features. The dimension of clinical and image features was resized into 11 and 64, respectively; and then concatenated together for final classification. We employed a Softmax activation function to activate the prediction value to ensure that the sum of prediction values of two classes (benign and malignant) equaled to 1. During evaluation, the class with larger prediction value is regarded as the classification result of the model, e.g. the prediction value [0.25, 0.75] means that proposed model predicts that the tumor is 25% benign and 75% malignant, and the final classification result is malignant.

**Visualization of the AI system:** For classification tasks on deep learning, Gradient-weighted Class Activation Mapping (Grad-CAM) was adopted to explain the performance of the proposed UC-STTNet.

For either tumor feature extraction block of the UC-STTNet, we defined the prediction score of each class as $y^{c}$, where $c$ means negative or positive categories. The output of the encoder is defined as $A^{k}$, where $k$ indicated the channel of $A$. As mentioned in [1], the importance of $A^{k}$ can be represented by the average value of the partial derivative of $y^{c}$of all parameters $A_{ij}^{k}$ in $A^{k}$, and it can be calculated by $a_{c}^{k}=\frac{1}{Z}\sum\sum\frac{\partial y^{c}}{\partial A_{ij}^{k}}$, where $z$ means the number of parameters in $A^{k}$. Finally, we used a weighted combination for the forward activation map and activated the result by ReLU function to get the visualization heatmap.

$$L_{Grad-CAM}^{c}=ReLU\left( \sum_{k} a_{c}^{k}A^{k} \right)$$

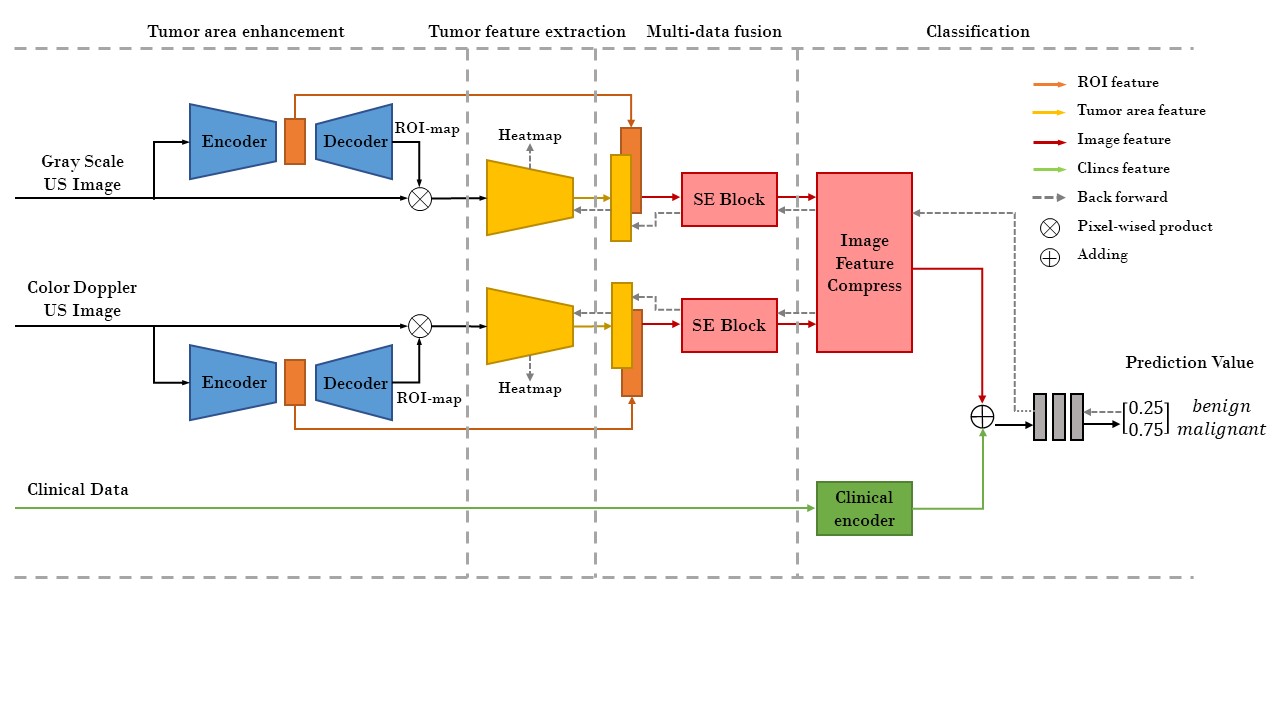


**Supplementary Figure 1. The network structure of the proposed UC-STTNet**

**Supplementary Table 1.** The detailed architecture of the proposed UC-STTNet

| **Classification** | FC-4096, FC-1024, FC-64 | Concatenation | FC-75, FC-35, FC-2, soft-max | |  | | | | | |
| --- | --- | --- | --- | --- | --- | --- | --- | --- | --- | --- |
|  | **Image Feature Compress** |  |  |  |  |  |  |  |  |  |
|  | FC-11 |  |  |  |  |  |  |  |  |  |
|  | **Clinical Encoder** |  |  |  |  |  |  |  |  |  |
| **Multi-data fusion** | Concatenation | SE layer with [4096, 256, 4096] channels for input, hidden and output | conv 1*1, 4096, avgpool |  | | | | | | |
| **Tumor feature extraction** | inceptionv3 block, 32*4, maxpool | inceptionv3 block, 64*4, maxpool | conv 3*3, 256, maxpool | conv 3*3, 512, maxpool | conv 3*3, 1024, maxpool |  | | | | |
| **Tumor area enhancement** | (conv 3*3, 32)*2, maxpool, (conv 3*3, 64)*2 | maxpool, (conv 3*3, 128)*2 | maxpool, (conv 3*3, 256)*2 | maxpool, (conv 3*3, 512)*2 | maxpool(2), (conv 3*3, 1024)*2 | Deconv 2*2, 32, stride 2, (conv 3*3, 32)*2, conv 3*3, 1 | Deconv 2*2, 64, stride 2, (conv 3*3, 64)*2 | Deconv 2*2, 128, stride 2, (conv 3*3, 128)*2 | Deconv 2*2, 256, stride 2, (conv 3*3, 256)*2 | Deconv 2*2, 512, stride 2, (conv 3*3, 512)*2 |
|  | **Encoder** | | | | | **Decoder** | | | | |

CONV: convolutional layer FC: fully connected layer**
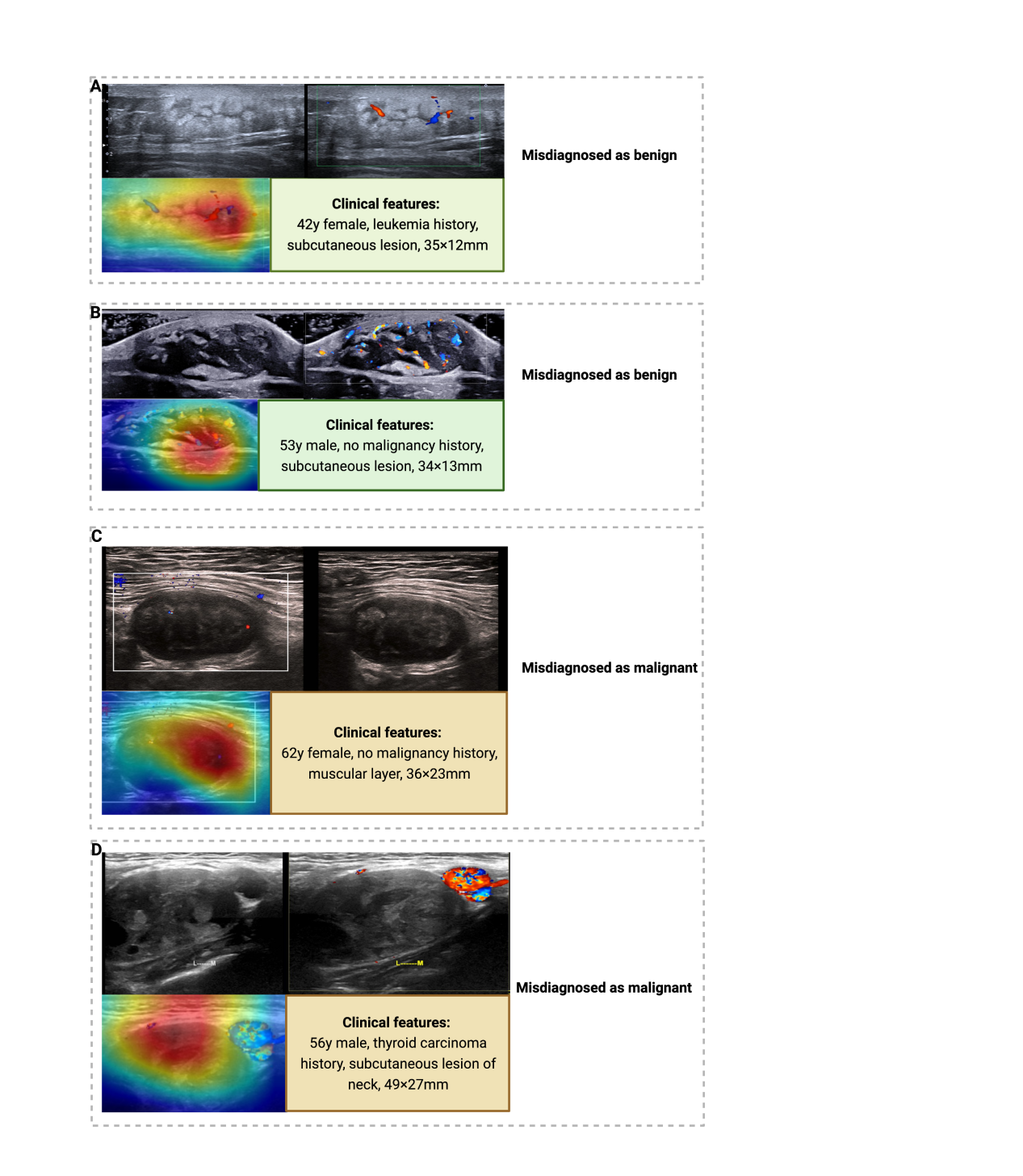
**

**Supplementary Figure 2. STT misdiagnosis cases of the AI system.**

2A. a subcutaneous tumor on the abdomen of a 42-year-old female with a size of 35×12mm. The patient had a history of leukemia. The AI system diagnosed it as a benign tumor and provided the heatmap identifying the correct active area. The lesion was diagnosed as a metastatic lesion of leukemia by pathology.

2B. a skin lesion on the chest of a 53-year-old male with a size of 34×13mm. The patient had no tumor or surgical history. The AI system diagnosed it as a benign tumor and provided the heatmap identifying the correct active area. The lesion was diagnosed as dermatofibrosarcoma protuberans (DFSP) by pathology. The DFSP lesion could be recognized through its skin changes.

2C. a STT mass in the muscular layer of the axilla of a 62-year-old female with a size of 36×23mm. The lesion has been found for four years. The patient had no tumor or surgical history. The AI system diagnosed it as a malignant tumor and provided the heatmap identifying the correct active area. The lesion was diagnosed as benign schwannoma by pathology.

2D. a subcutaneous tumor on the left neck of a 46-year-old male with a size of 49×27mm. The patient had thyroid papillary carcinoma 2 years ago. The AI system diagnosed it as a malignant tumor and provided the heatmap identifying the correct active area. The lesion was diagnosed as benign schwannoma by pathology.
